# Editorial: Advanced three-dimensional platforms for tissue regeneration: when the microenvironment matters

**DOI:** 10.3389/fbioe.2023.1265642

**Published:** 2023-08-09

**Authors:** Enrico Domenico Lemma, Antonella Motta, Alessio Bucciarelli, Pamela Mozetic

**Affiliations:** ^1^ Department of Engineering, Università Campus Bio-Medico di Roma, Rome, Italy; ^2^ Department of Industrial Engineering, University of Trento, Trento, Italy; ^3^ Laboratorio RAMSES, IRCSS Istituto Ortopedico Rizzoli, Bologna, Italy; ^4^ Institute of Nanotechnology (NANOTEC), National Research Council, Lecce, Italy

**Keywords:** 3D cell culture, microfabrication, tissue engineering, 3D printing, direct laser writing, cell niche, 3D scaffolding

Several studies have demonstrated that cells closely interact with their surroundings, not only in physiological conditions but also within artificial microenvironments. Indeed the microenvironment exerts control of cell behavior and function in health and disease (Clark and Vignjevic, 2015; Young et al., 2016; Rais et al., 2023). The major player in the cellular microenvironment is represented by the extracellular matrix (ECM), which acts as a structural foundation for cells, providing them with biochemical and biophysical cues (Urbanczyk et al., 2020; Eichinger et al., 2021). Progress in the development of functional and biomimetic materials in the field of tissue engineering has led to improved three-dimensional (3D) scaffolds and *in vitro* models. These are often 3D structures or supportive materials hosting organoids or (stem) cell colonies and are powerful resources for studying the behavior of single cells, cell-cell interactions, and the interplay between cells and materials (Cadamuro et al., 2023). In addition, it is generally acknowledged that three-dimensionality, together with suitable biochemical cues, also plays a key role in inducing cells to develop organized structures resembling native tissues. Therefore, several approaches have been pursued to design 3D structures mimicking the physiological conditions in which cells exert their functions. Yet, the reconstruction of a fully functional 3D model of tissues and organs is far from being achieved. Difficulties arise not only from technological challenges but also from the poor understanding of several basic mechanisms underlying the successful organization of (stem) cells toward complex (e.g., multi-layered) patterns, which limit the potential of 3D micro-scaffolds as sources for regenerating tissues.

Here, we compiled a collection of original research and review papers providing the reader with an overview of the salient achievements in the field of 3D microenvironments, broadly intended as synthetic or naturally-derived, variously fabricated, and functionalized milieus.

In particular, four review papers outline the state of the art in the respective field of expertise of the authors. The reciprocity between cells and extracellular matrix (ECM) is deeply studied in the review by Urciuolo et al., where strategies for replicating simplified models of the tissue microenvironment by constructive (e.g., microfabrication) and disruptive (e.g., decellularization) methods are shown. Moreover, the authors give an extensive description of scaffold design evolution by comparing exogenous scaffold fabrications and endogenous-based approaches, highlighting the higher physiologically relevant environment of the endogenous engineered tissues.

Gisone et al. explore the main approaches used to establish *in vitro* models for cardiac tissue engineering, emphasizing the role of IPS-derived cardiomyocytes as cellular models. The authors extensively discuss the advantages of 3D *in vitro* heart models, with reference to scaffold-free and scaffold-based approaches for regeneration. Ransanz et al., instead, focus on brain neural cell models, highlighting the importance of mechanical cues in the artificial microenvironment (e.g., stiffness, viscosity, static, and dynamic cues) for migration, proliferation, and differentiation of neural stem cells. The authors highlight the role of microengineering to recreate 3D tissue-like structures for scaffold-based neuromechanobiology applications. A detailed overview of fabrication technologies and characterization tools is also provided. A fundamental aspect in the study of 3D modeling for tissue engineering is the complex physico-chemical interplay between cells and the surrounding microenvironment, which is the main Research Topic of the review by Bruschi et al. The authors focus on methods for engineering the hematopoietic niche, providing an overview of the strategies to identify the biophysical factors and biochemical stimuli pivotal for the controlled growth and differentiation of hematopoietic stem cells.

The basic role played by ECM proteins in artificial microenvironments is demonstrated in the original research manuscript by Lemma et al. Here, a systematic study is performed to show that the density of ECM protein (specifically, fibronectin) molecules is a predominant factor in determining cell spreading, and cells are able to sense FN density on substrates and adjust their spreading area according to the number of available binding sites, whether these are homogeneously distributed or organized in geometrical patterns. Moreover, this behavior is mainly driven by the β1 integrin subunit, with other FA proteins playing a secondary role. To achieve the aforementioned results, several patterns were fabricated using two-photon lithography, a direct writing laser technique also used by Sharaf et al., who present innovative cage-like scaffolds decorated with micro-pillars to show enhanced primary microglia colonization and branching compared to smooth structures. The importance of technology advancements and refinements in fabricating 3D microenvironments for cell culture is presented by Jeršovaitė et al., who investigate the optimal post-processing to minimize the residual presence of unpolymerized, toxic monomers in 3D-printed microporous scaffolds. The authors demonstrate that the improved degree of crosslinking significantly affects scaffold biocompatibility and osteogenic differentiation of mesenchymal stem cells from rat dental pulp. Finally, Dupard et al. also exploit a 3D-printing technique, namely, fused deposition modeling, to develop an innovative bioreactor for the maintenance of human hematopoietic stem cells.

In conclusion, this Research Topic collects a number of interesting contributions summarizing the importance of recapitulating ECM traits in the design of novel platforms for 3D cell culture and tissue regeneration. The reported studies contribute to shed light on the intimate yet complex interaction of chemical, biological, and mechanical factors, which characterize living tissues.
